# Atom-economical group-transfer reactions with hypervalent iodine compounds

**DOI:** 10.3762/bjoc.14.108

**Published:** 2018-05-30

**Authors:** Andreas Boelke, Peter Finkbeiner, Boris J Nachtsheim

**Affiliations:** 1 Institute for Organic and Analytical Chemistry, University of Bremen, 28359 Bremen, Germany

**Keywords:** atom economy, benziodoxolones, homogeneous catalysis, hypervalent iodine, iodonium salts

## Abstract

Hypervalent iodine compounds, in particular aryl-λ^3^-iodanes, have been used extensively as electrophilic group-transfer reagents. Even though these compounds are superior substrates in terms of reactivity and stability, their utilization is accompanied by stoichiometric amounts of an aryl iodide as waste. This highly nonpolar side product can be tedious to separate from the desired target molecules and significantly reduces the overall atom efficiency of these transformations. In this short review, we want to give a brief summary of recently developed methods, in which this arising former waste is used as an additional reagent in cascade transformations to generate multiple substituted products in one step and with high atom efficiency.

## Introduction

Atom economy (AE) is an important parameter which helps to evaluate the overall efficiency of a chemical reaction or a chemical process [1–2]. It is defined as the quotient between the molecular mass of the desired reaction product(s) and the molecular mass of all reactants (Equation 1):

[1]
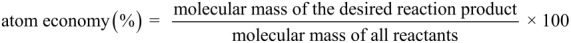


**Scheme 1:**
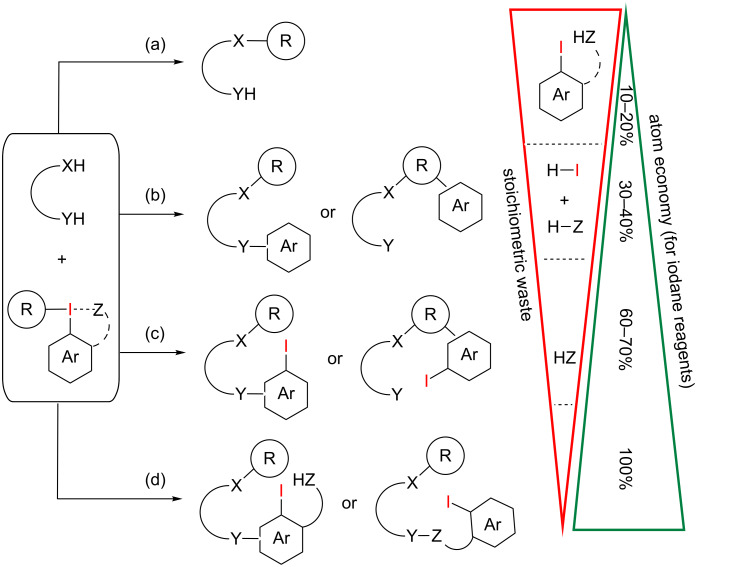
Overview of different types of iodane-based group-transfer reactions and their atom economy based on the molecular mass of the corresponding λ^3^-iodane.

In an ideal reaction with 100% atom economy, every atom of the reactants is becoming part of the desired product. In this short review, we want to discuss recent advances in atom-economical transformations using hypervalent iodine reagents (iodanes) as electrophilic group-transfer reagents. Iodanes, in particular iodonium salts, are well-balanced reagents in terms of stability, reactivity and synthetic and/or commercial availability and therefore it is not surprising to see these compounds as key reagents in a great number of recently developed transformations [3–15]. However, in terms of atom economy, they have an intrinsic problem: their high reactivity is based on the emergence of aryl iodides as supernucleofuges. λ^3^-Iodanes are the generally preferred hypervalent iodine compounds for electrophilic group transfer reactions, during which the central iodine atom is transformed from a high energy hypervalent state into a normal valent state by a two-electron reduction. The high stability of the newly formed aryl iodide is the thermodynamic driving force for all λ^3^-iodane-mediated oxidative transformations. Even though this process guarantees the high reactivity of these reagents, it has one major obstacle: after the oxidation process, stoichiometric amounts of the aryl iodide are produced as waste. Aryl iodides, as nonpolar organic waste, are oftentimes hard to separate from the desired reaction products since they cannot be simply washed out with aqueous solutions. Instead, they must be separated by column chromatography. In terms of AE, the high atomic mass of iodine (126.9 u) leads to a dramatic negative impact of iodanes on this “green” reaction parameter. To overcome this obstacle, promising approaches are the use of iodoarenes as precatalysts in combination with external co-oxidants and the utilization of specific hypervalent iodine compounds (polymer-supported as well as non-polymeric species), whose reduced forms are easy to recycle [3–4,16–17]. On the other hand, the additional incorporation of the former iodoarene waste into the reaction product via a cascade reaction does not only improve the overall AE of a chemical reaction but also directly leads to highly functionalized target molecules. A rough estimate about the impact of the incorporation efficiency of the iodane on the AE is depicted in Scheme 1. Here, only the molecular mass of the corresponding iodane substrates is taken into account for AE estimation.

The transfer of only one functional group, for instance in iodane-mediated electrophilic monoarylations, produces not only the aryl iodide, but also stoichiometric amounts of salt side-products, limiting the AE of these transformations to roughly 10–20% (Scheme 1, reaction (a)). In the second case, both organic residues (one carbon or heteroatom ligand plus the arene of the former aryl-λ^3^-iodane or aryliodonium salt) are transferred to the substrate (Scheme 1, reaction (b)). The loss of iodine and the counterion still limits the overall AE to 30–40%. A dramatic increase in AE arises, if the iodine atom is incorporated in the desired reaction product as well (Scheme 1, reaction (c)). This does not only lead to an overall AE of up to 70% but also produces synthetically versatile intermediates for subsequent transformations, in particular metal-catalyzed cross-coupling reactions. If benziodoxolones or benziodoxoles are used as group-transfer reagents, nearly 100% AE is possible since the counterion (the carboxylate or alcoholate) is covalently attached to the aryl iodide and hence does not get lost during the reaction cascade (Scheme 1, reaction (d)). This is very effective, if this *ortho*-functionality is a desired part of the final reaction product or if it can be readily transformed into synthetically useful functional groups.

In this short review, we will give a brief summary of very recent efforts toward the atom-economical use of aryl-λ^3^-iodanes, in particular aryliodonium salts in group-transfer reactions. In our definition, this includes transformations in which at least two of the three ligands attached to the iodane are part of the target molecule or in which the iodane acts as an oxidant and a group-transfer reagent in a consecutive reaction sequence. The chapters are divided by the structure of the transferred functional groups, starting from simple diarylations and oxidative arylations with moderate AE to highly atom efficient transformations using alkynyl and azide-substituted benziodoxolones. The given AE values are simplified and were calculated on the basis of the key substrates, whereas the required equivalents of all starting materials (iodane and usually its reaction partner) are taken into account. Other additives, such as additional bases, acids or catalysts were neglected.

## Review

### 1. Diaryliodonium salts

#### 1.1. Acyclic diaryliodonium salts

**Scheme 2:**
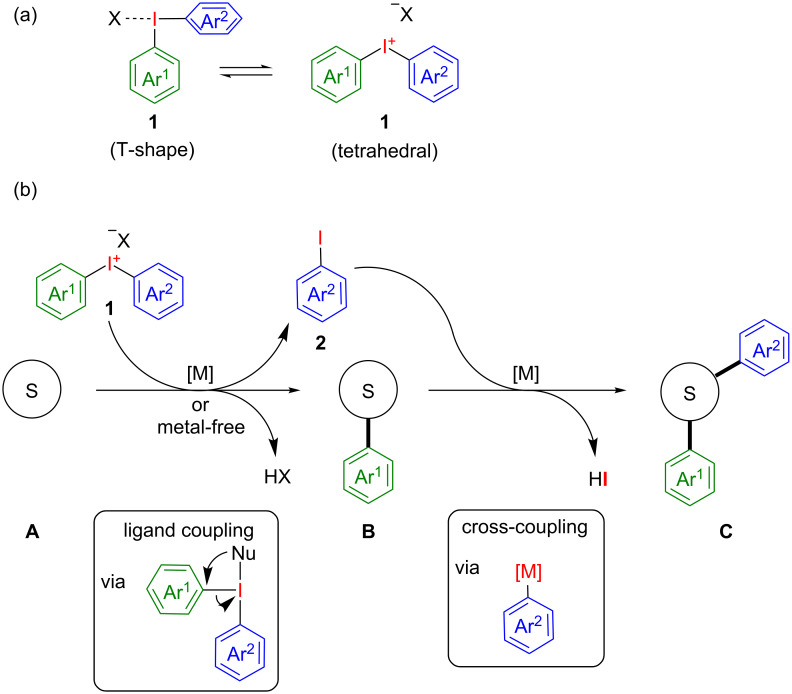
(a) Structure of diaryliodonium salts **1**. (b) Diarylation of a suitable substrate **A** with one equivalent of diaryliodonium salt **1**.

Acyclic diaryliodonium salts **1** find widespread application in numerous metal-free and transition metal-mediated electrophilic arylation protocols [18–22]. While in solid state they clearly have a T-shaped pseudotrigonal bipyramid structure, the common structural motif for λ^3^-iodanes, recent theoretical investigations revealed a potential role of the cationic, normal valent tetrahedral form, in atom transfer processes (Scheme 2a) [23]. Throughout this review, iodonium salts will be shown consequently in the latter structure due to clarity and due to literature habits.

Typically, only one of the two aryl ligands is transferred to the substrate, yielding a monoarylated reaction product and aryl iodide as stoichiometric waste. Examples for their atom-economical utilization, in which at least both aryl ligands are transferred, are still rare. A general approach would involve at first a metal-catalysed or metal-free arylation step of a suitable substrate **A** with the diaryliodonium salt **1** to give monoarylated intermediate **B**. Subsequently, a metal-catalysed cross-coupling initiated by an oxidative addition of the metal catalyst into the C–I bond of the emerging iodoarene **2** affords the diarylated product **C** (Scheme 2b).

**Scheme 3:**
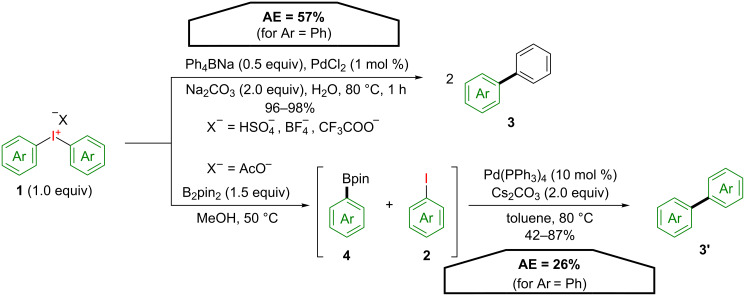
Synthesis of biphenyls **3** and **3’** with symmetrical diaryliodonium salts **1**.

A first report utilizing this strategy was published by Bumagin and co-workers as early as 1995 [24]. Here, symmetrical diaryliodonium salts **1** were used in a palladium-catalysed cross-coupling reaction with sodium tetraphenylborate (Scheme 3). This reaction not only provides excellent yields of the respective biphenyls **3** but also exhibits a high AE (57% for Ar = Ph). However, its general synthetic utility is limited since it requires highly reactive boron compounds as nucleophiles.

Symmetrical biphenyls **3’** can be generated from the corresponding symmetrically substituted diaryliodonium salts **1** and bis(pinacolato)diboron as demonstrated by Muñiz and co-workers [25]. In the first step, a mild carbon–boron bond formation gives one equivalent of arylboronic ester **4** and an iodoarene **2** through a metal-free boron arylation. Subsequent cross coupling under Suzuki conditions affords symmetrical biphenyls **3’** in good yields. Due to the temporary introduction and cleavage of the boron moiety the formal atom economy for this transformation is rather low (26% for Ar = Ph). However, 45% of the molecular weight of the starting iodonium salt **1** is present in the product **3’**.

**Scheme 4:**
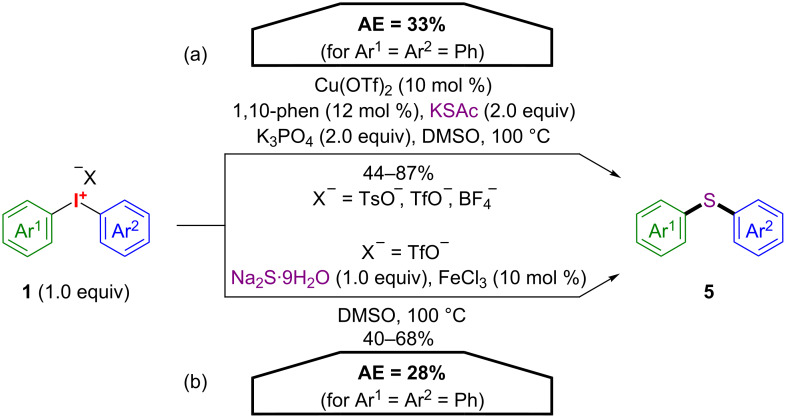
Synthesis of diaryl thioethers **5**.

Diaryl thioethers **5** can be synthesized using either cyclic iodonium salts (will be discussed briefly in section 1.2) or their acyclic counterparts **1** (Scheme 4) [26–27]. Jiang and co-workers developed a Cu(II)-catalysed methodology for the conversion of acyclic diaryliodonium salts **1** and potassium thioacetate to the corresponding thioethers **5** (pathway (a)) and later applied the optimized reaction conditions towards cyclic iodonium salts. In an independent work, Li and co-workers successfully utilized a catalytic system based on FeCl_3_ with comparable results (pathway (b)). However, both procedures have only moderate AE values (28–33% for Ar^1^ = Ar^2^ = Ph), since the transferred aryl groups account for only about 35% to the mass of the respective diaryliodonium salts **1**.

**Scheme 5:**
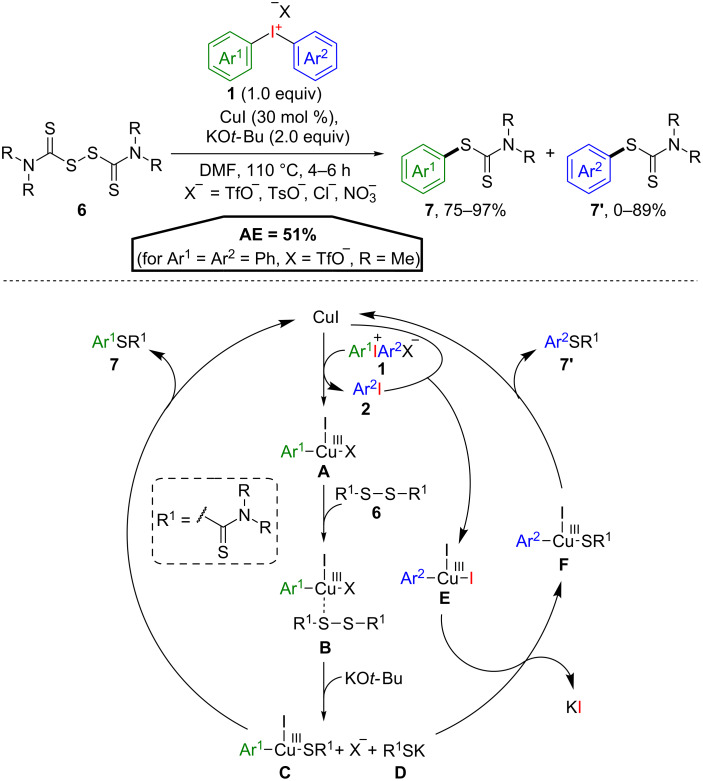
Synthesis of two distinct *S*-aryl dithiocarbamates **7** and **7’** from one equivalent of diaryliodonium salt **1**.

An atom efficient *S*-arylation of tetraalkylthiuram disulfides **6** was developed by Dong and co-workers under Cu(I)-catalysis (Scheme 5) [28]. This reaction yields two equivalents of *S*-aryl dithiocarbamates **7** and **7’** in typically high yields applying only one equivalent of a diaryliodonium salt **1**. It is worth to mention, that the AE of 51% (for Ar^1^ = Ar^2^ = Ph, X = TfO^–^, R = Me) is only valid if both dithiocarbamates **7** and **7’** are defined as desired reaction products. If symmetrical diaryliodonium salts are used, as also demonstrated, only one arylation product is formed. The proposed reaction mechanism starts with the oxidative addition of the diaryliodonium salt **1** to copper(I) iodide affording the Cu(III) species **A**, releasing aryl iodide **2**. Coordination of the disulfide **6** to the metal centre leads to complex **B**, followed by the base-induced formation of arylcopper sulfide complex **C** and potassium salt **D** upon treatment with KO*t-*Bu. Reductive elimination from **C** produces the product **7**, under regeneration of the Cu(I) species. The released aryl iodide **2** on the other hand then undergoes oxidative addition forming the Cu(III) species **E**, which can provide intermediate **F** upon reaction with the potassium salt **D** and the release of potassium iodide. Reductive elimination then affords the second *S*-aryl dithiocarbamate **7’**.

**Scheme 6:**
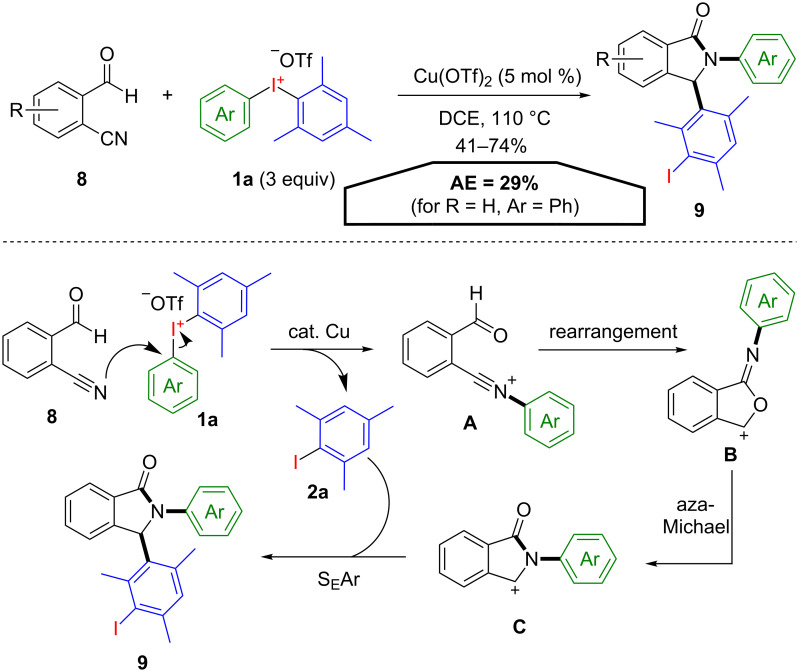
Synthesis of substituted isoindolin-1-ones **9** from 2-formylbenzonitrile **8** and the postulated reaction mechanism.

If both aryl groups are not transferred to the same reaction centre in metal-mediated diarylations when unsymmetrical diaryliodonium salts are used as aryl-transfer reagents, poor chemoselectivies are often observed. Since electronic differences in many cases exhibit only unsatisfactory levels of selectivity [29–30], sterically demanding groups, such as mesityl moieties, are mostly used to solve this issue. However, the high steric demand can be of disadvantage if the development of an atom-economical transformation is intended through a subsequent functionalization of the emerging, now less reactive, mesityl iodide. A procedure which impressively combines the selectivity of mesityl(aryl)iodonium salts **1a** with this difficult second arylation step was developed by Li and co-workers [31]. Under copper-catalysis diarylated isoindolin-1-ones **9** were formed starting from 2-formylbenzonitriles **8** upon treatment with mesityl(aryl)iodonium salts **1a** (Scheme 6). A plausible reaction mechanism starts with the selective terminal arylation of the nitrile, forming the arylnitrilium cation **A** and 2-iodomesitylene (**2a**). Intramolecular cyclization leads to the iminofuranylium intermediate **B** which affords the cationic isoindolin-1-one structure **C** via subsequent intramolecular aza-Michael type rearrangement. This intermediate reacts in the final step with the electron-rich 2-iodomesitylene (**2a**) in an S_E_Ar reaction to give the diarylated isoindolin-1-one **9**. A drawback of these reaction conditions is the use of excess (3 equiv) mesityl(aryl)iodonium salt **1a** to achieve satisfying yields. This dramatically lowers the atom-economy to only 29% (for R = H, Ar = Ph), instead of theoretical 75%, if only one equivalent of the iodane **1a** would have been sufficient.

**Scheme 7:**
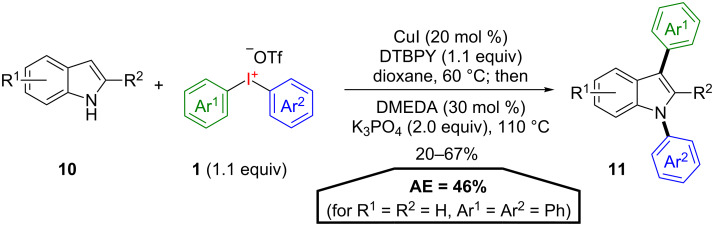
Domino C-/N-arylation of indoles **10**.

In recent years a variety of domino C–H and/or N–H arylations as powerful tools towards selective diarylations of (hetero)arenes was employed by Greaney and co-workers, who were among the first to establish more atom-economical procedures with λ^3^-iodanes. These methods commonly exhibit good AE for (di)arylation procedures, since only small excesses of diaryliodonium salts are necessary. In an initial study, the selective diarylation of indoles **10** using symmetrical and unsymmetrical diaryliodonium salts **1** was demonstrated (Scheme 7) [32]. A selective C–H arylation at C3 of the indole was realised under copper catalysis before the addition of a ligand and an inorganic base initiated the N-arylation with the in situ formed iodoarene. The desired diarylated indoles **11** are obtained with an AE of 46% (for R^1^ = R^2^ = H, Ar^1^ = Ar^2^ = Ph). The scope of this transformation is broad, only C2-substituted indoles show poor reactivity. Furthermore, this is a rare example for good chemoselectivities in atom-efficient reaction cascades even if unsymmetrical substituted diaryliodonium salts are applied.

**Scheme 8:**
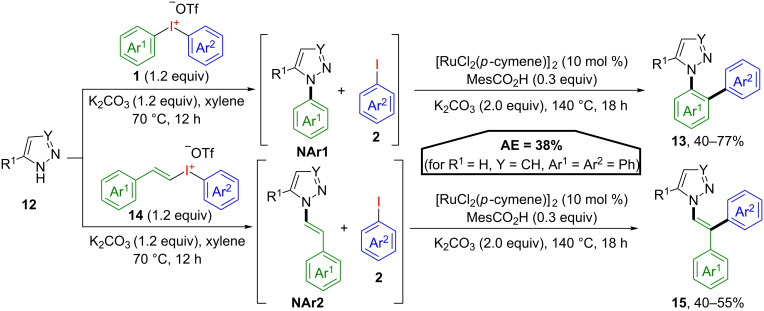
Domino modification of N-heterocycles **12** via in situ-generated directing groups.

Based on this initial procedure, an impressive atom-economical biphenylation of N-heterocycles was developed [33]. This method involved a direct N-arylation of pyrazoles or triazoles **12** under basic conditions, followed by a ruthenium-catalysed C–H arylation with the emerging aryl iodide (Scheme 8). Due to the fact that the first step of this reaction sequence proceeds under metal-free conditions, the selective transfer of the more electron-deficient aryl group of the unsymmetrical iodonium salts **1** is achieved. The N*-*arylated heterocycle **NAr1** then serves as the directing group in the following *ortho*-C–H functionalisation with the iodoarene as coupling reagent to yield biphenyls **13**. The optimized reaction conditions are also applicable to alkenyl(aryl)iodonium salts **14** making arylated benzylidenes **15** accessible through the N-alkenylated intermediate **NAr2**.

The AE for this transformation is 38% (for R^1^ = H, Y = CH, Ar^1^ = Ar^2^ = Ph), a typical value for iodane-mediated double functionalisations with aryl iodonium salts.

**Scheme 9:**
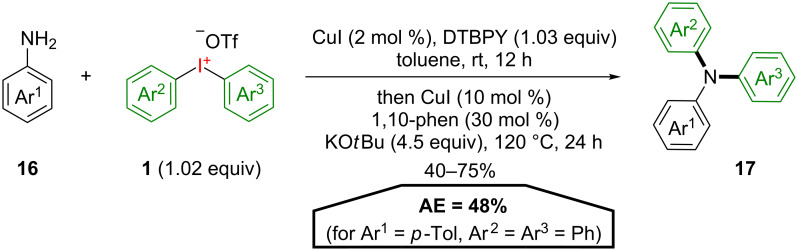
Synthesis of triarylamines **17** through a double arylation of anilines.

Another achievement of Greaney and co-workers is the atom-economical double arylation of anilines **16** with symmetrical diaryliodonium salts **1** (Scheme 9) [34]. The corresponding triarylamines **17** are important structural motifs for organic electronics and can be obtained in good yields and an AE of 48% (Ar^1^ = *p*-Tol, Ar^2^ = Ar^3^ = Ph) using this Cu(I)-catalysed domino approach. The formation of an intermediately found diarylaniline supposedly follows a Cu(I)/Cu(III) cycle, whereas a radical mechanism is proposed for the second arylation. Unfortunately, a selective aryl transfer using unsymmetrical diaryliodonium salts was not successful under the reaction conditions, giving only mixtures of mono N-arylated anilines in low yields after the first step.

**Scheme 10:**
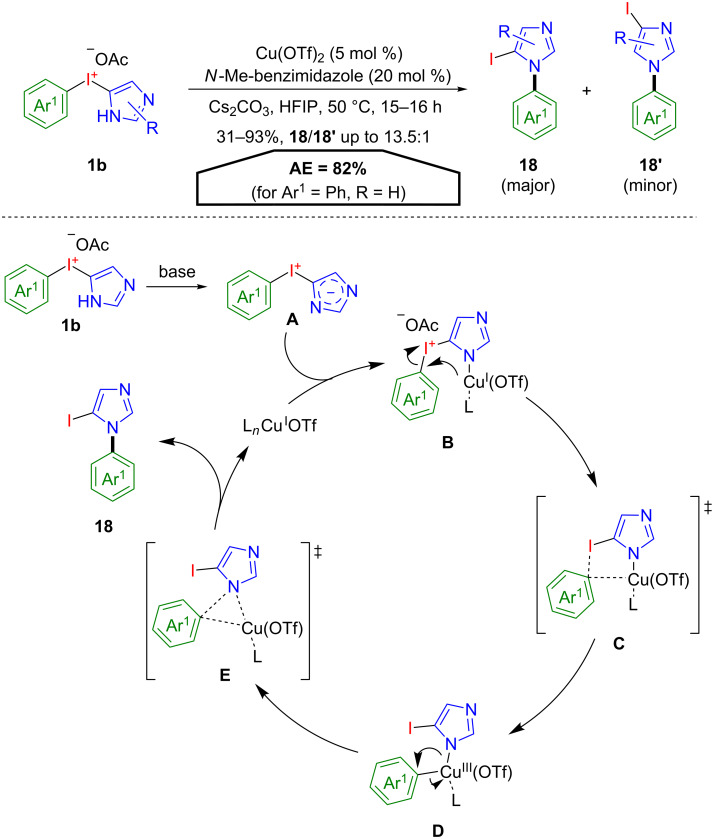
Selective conversion of novel aryl(imidazolyl)iodonium salts **1b** to 1,5-disubstituted imidazoles **18**.

A different approach towards the functionalisation of heterocycles was taken by Shafir, Lledós, and co-workers, who investigated the intrinsic conversion of novel N-heterocyclic aryliodonium salts **1b** [35]. Under the influence of base, copper catalyst and *N*-Me-benzimidazole as a ligand, the heterocyclic aryliodonium salt **1b** was converted into the *N*1-aryl-5-iodoimidazole **18** as the major isomer (Scheme 10). Compound **18** is a valuable intermediate for a variety of coupling reactions to yield 1,5-disubstituted imidazoles – structural motifs which are generally difficult to obtain – in a selective fashion. The proposed reaction mechanism based on DFT calculations starts with the deprotonation of the imidazolyl group to give betain **A**, which binds to a Cu(I)-OTf fragment leading to complex **B**. Next, the aryl group is transferred from the iodine to the copper atom leading to the Cu(III) complex **D** via a five-membered transition state **C**. Reductive elimination through transition state **E** provides **18** and restores the active Cu(I) species. Since this reaction follows an intramolecular pathway, it is featured by a remarkable high atom economy of 82% (for Ar^1^ = Ph, R = H), lowered only by the loss of one formal equivalent of acetic acid.

#### 1.2. Cyclic diaryliodonium salts

**Scheme 11:**
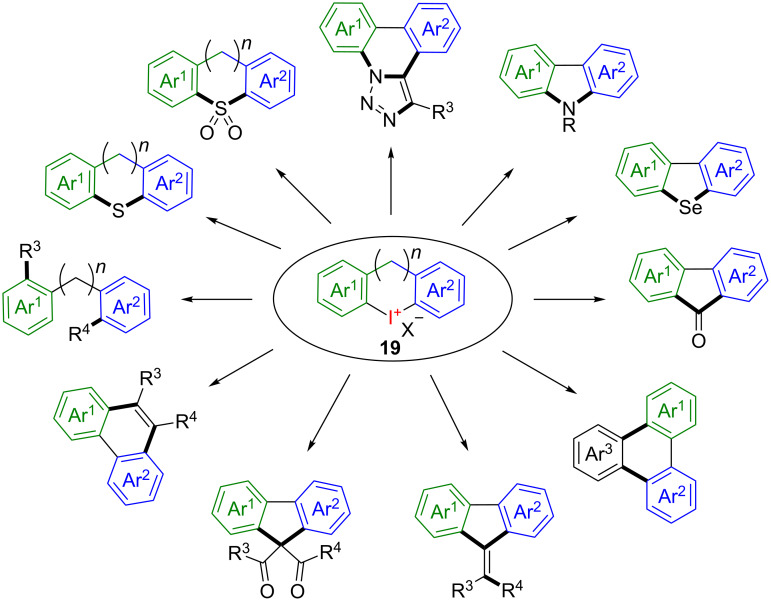
Selected examples for the application of cyclic diaryliodonium salts **19**.

Within the vast scope of hypervalent iodine compounds the class of cyclic diaryliodonium salts **19** is predestined to undergo atom-efficient transformations. Owing to their unique structure in which both carbon ligands attached to the central iodine atom are connected to one another, no iodoarene “waste” can be formed in the reaction process. Depending on the reaction conditions the selective functionalisation of just one aryl iodine moiety or the modification of both carbon ligands in either a symmetrical or unsymmetrical transformation is possible leading to a plethora of useful carbo- and heterocycles (Scheme 11).

The chemistry of cyclic diaryliodonium salts has been reviewed by Grushin in 2000 [36] and more recently by Goswami and co-worker [37]. Therefore, this topic will not be discussed in further detail in this article. However, since iodine and the counterion are inevitable stoichiometric side products, it is worth mentioning that the theoretical AE for these particular transformations is usually limited to 30–40%.

### 2. (Dicarboxyiodo)benzenes

From a synthetic point of view, (dicarboxyiodo)benzenes **20** are probably one of the most popular hypervalent iodine compounds in organic synthesis [3,9,38–41]. They have been widely applied in C–H oxygenations, nitrene generations, oxidative dearomatisations and dehydrogenative couplings by transferring one of their two carboxyl ligands or external oxygen nucleophiles to a substrate. In all these “classical” reactions aryl iodides are produced as stoichiometric waste products once again. The further utilization of this side product in a subsequent reaction step would dramatically increase the AE for such oxidative transformations.

**Scheme 12:**
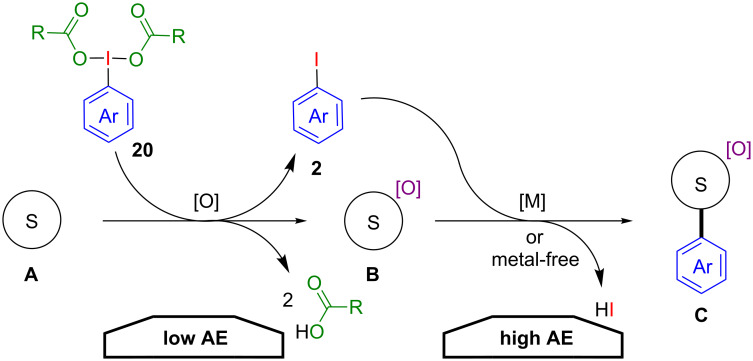
Tandem oxidation–arylation sequence with (dicarboxyiodo)benzenes **20**.

A general procedure for such a tandem sequence would involve an initial oxidation step of a substrate **A** followed by a coupling of the formed iodoarene **2** with the oxidised substrate **B** either under metal-free or metal-catalysed conditions to form the product **C** (Scheme 12).

**Scheme 13:**
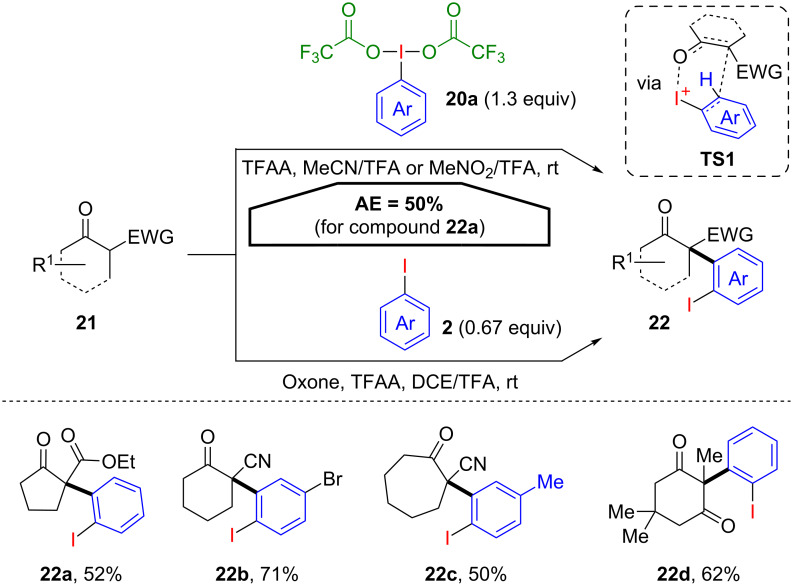
Oxidative α-arylation via the transfer of an intact 2-iodoaryl group.

The first examples for an oxidation–arylation cascade using (dicarboxyiodo)benzenes were reported by Shafir and co-workers [42–43]. Herein, the authors describe an efficient α-arylation of a variety of 1,3-dicarbonyl derivatives **21** using [bis(trifluoroacetoxy)iodo]benzene (**20a**, PIFA). In this metal-free approach the target structures **22** are efficiently synthesised even without any initial prefunctionalisation of the arene moiety (Scheme 13). Remarkably, the intact 2-iodoaryl group is transferred via presumed transition state **TS1**, which leads to a good AE (50% for **22a**) and allows the further transformation of the α-arylation product **22** via cross coupling reactions. In addition, the in situ generation of the PIFA reagent proved viable, in order to temporary generate potentially unstable λ^3^-iodane derivatives and immediately convert them under the established α-arylation conditions.

**Scheme 14:**
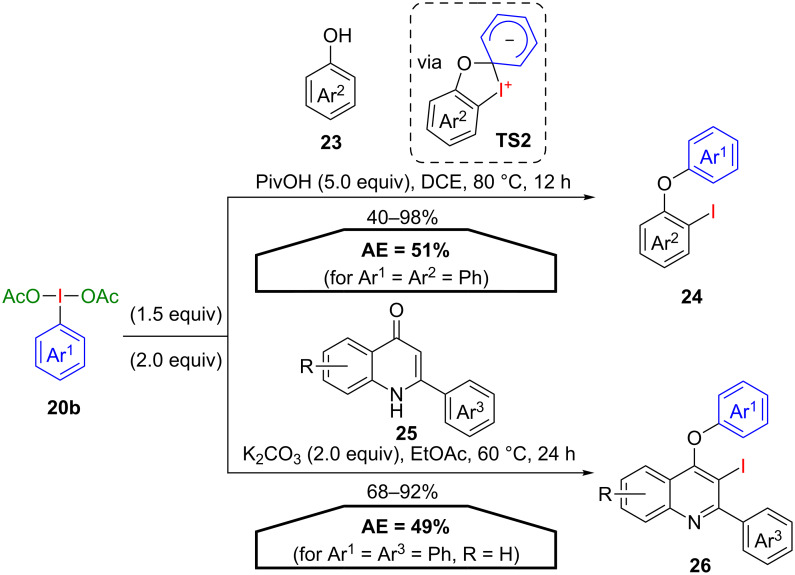
Tandem *ortho*-iodination/O-arylation cascade with PIDA derivatives **20b**.

A PIDA ((diacetoxyiodo)benzene)-mediated *ortho*-iodination/O-arylation cascade was developed by Panda and co-workers using diversely functionalised phenols **23** (Scheme 14) [44]. The reaction presumably proceeds via an *ortho*-addition of the λ^3^-iodane **20b**, followed by an iodine-to-oxygen 1,3-aryl migration via a concerted mechanism through the five-membered intermediate **TS2** to afford the corresponding diaryl ether **24**. A mechanistically similar *ortho*-iodination/O-arylation cascade for the modification of 2-arylquinolones **25** was developed by Xie and co-workers, providing the respective products **26** in a one pot sequence [45]. Both methods are characterised by a good AE (49–51% for Ar = Ph) due to the incorporation of both, the aryl group and the iodine atom. However, the usage of 1.5 to 2.0 equivalents of the hypervalent iodine reagent **20b** lowers the conclusive atom efficiencies.

**Scheme 15:**
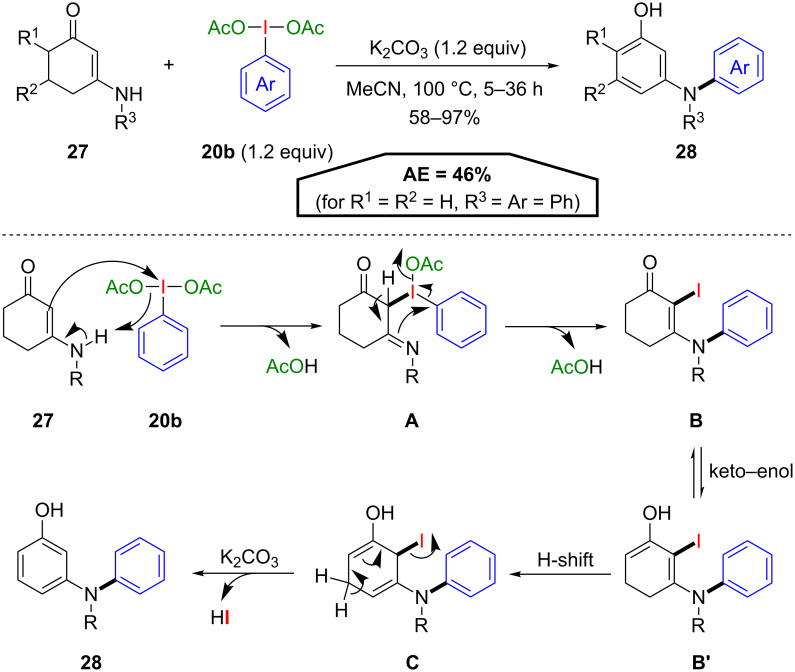
Synthesis of *meta*-*N*,*N*-diarylaminophenols **28** and the postulated mechanism.

In another closely related procedure, Das and co-workers employed PIDA derivatives **20b** for an aromatization–arylation cascade of exocyclic β-enaminones **27** [46]. Under basic reaction conditions *meta*-*N*,*N*-diarylaminophenols **28** were obtained in good to excellent yields using **20b** both as the aromatization and arylation agent (Scheme 15). In the first step of the postulated mechanism, a ligand exchange of the starting material **27** and PIDA **20b** forms intermediate **A**, which leads to the formation of the stable N-arylated intermediate **B** via an iodine-to-nitrogen 1,3-phenyl migration. An equilibrium with its enol form **B’** is proposed, which affords the aromatization product **28** via H-shift to intermediate **C** and subsequent dehydroiodination under basic conditions. Although the iodine atom is not incorporated into the final product, the calculated AE (46% for R^1^ = R^2^ = H, R^3^ = Ar = Ph) is close to the procedures of Panda and Xie due to lower PIDA loadings.

**Scheme 16:**
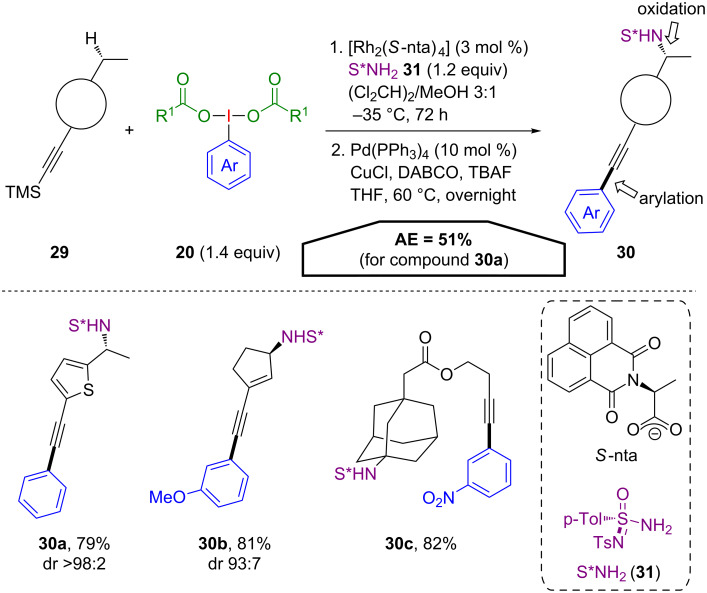
(Dicarboxyiodo)benzene-mediated metal-catalysed C**–**H amination and arylation.

A metal-catalysed procedure for the atom-economical utilization of (dicarboxyiodo)benzenes **20** in a tandem C(sp^3^)–H amination/sila-Sonogashira–Hagihara reaction was developed by Dauban and co-workers (Scheme 16) [47]. The first step of this sequence includes an iodine(III)-mediated rhodium-catalysed enantioselective amination of an unactivated C(sp^3^)–H bond with a chiral sulfonimidamide **31**. Afterwards, the iodoarene byproduct of the first step is coupled with the alkyne under palladium catalysis. A broad range of TMS-substituted acetylenes **29** was converted efficiently under the optimized reaction conditions, forming the desired arylacetylenes **30** in good yields with high stereoselectivity. The applicable substrate scope is rather broad including thiophene, cyclopentene and adamantyl derivatives **30a**–**c** with good AE values (51% for **30a**).

**Scheme 17:**
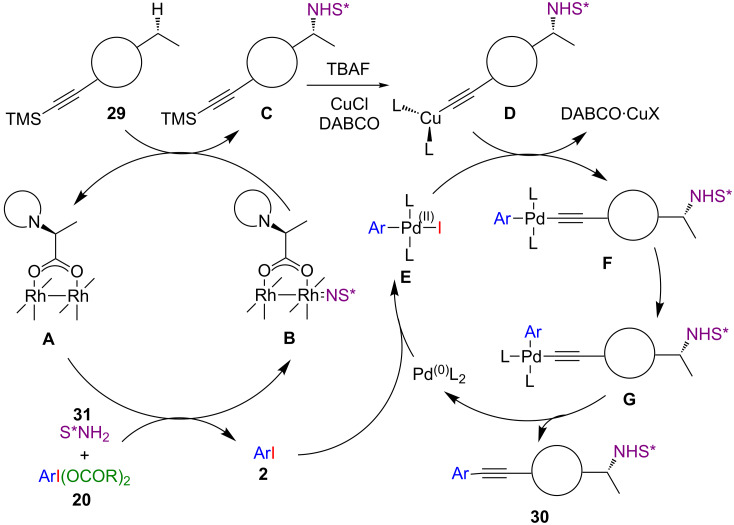
Postulated mechanism for the amination–arylation sequence.

The proposed mechanism involves two distinct catalytic cycles for the amination and the arylation reaction step (Scheme 17). First, a metallanitrene **B** is formed via the reaction of the (dicarboxyiodo)benzene **20** with the sulfonimidamide **31** in the presence of the chiral rhodium(II) catalyst **A**. Hereby, one equivalent of iodoarene **2** is released. The insertion of the nitrene species into the C(sp^3^)–H bond affords the amination product **C**, which is the final product of the first reaction step. In the following step, **C** is subsequently desilylated with TBAF in the presence of CuCl and DABCO to obtain the alkynylcopper species **D**. In the meantime oxidative addition of the previously released iodoarene **2** to the Pd(0) species occurs and the resulting palladium(II) complex **E** then undergoes transmetallation with the copper species **D** to provide complex **F**. Isomerisation to the *cis*-palladium complex **G** and subsequent reductive elimination finally delivers **30**.

**Scheme 18:**
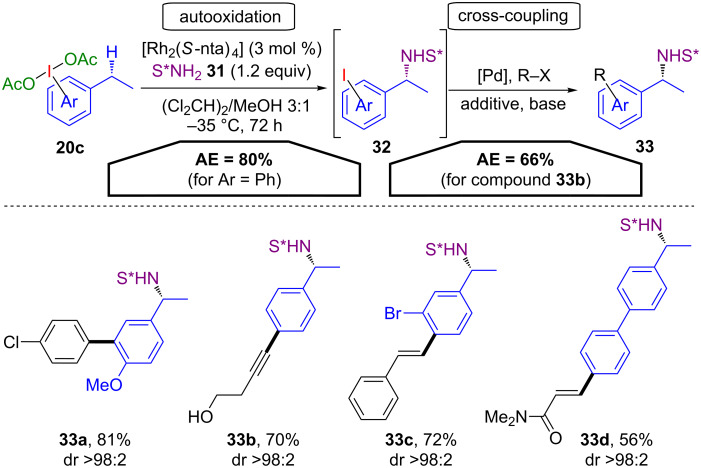
Auto-amination and cross-coupling of PIDA derivatives **20c**.

In such sequences, the iodane can also be prone to autooxidation processes, as demonstrated by Dauban and co-workers. Alkyl-substituted PIDA derivatives **20c** are the substrates for both the chiral C(sp^3^)–H amination and for the following cross-coupling reaction (Scheme 18) [48]. In the first step, the C(sp^3^)–H bonds of PIDA derivatives were efficiently converted via an auto-amination with the external sulfonimidamide **31** to form the corresponding aminated iodoarenes **32**. These species smoothly underwent subsequent Suzuki–Miyaura, Sonogashira and Mirozoki–Heck couplings. The respective arylated, alkynylated and alkenylated products **33a**–**d** were obtained in high yields with excellent stereoselectivity. Due to the intramolecular pathway of the first step, high AE values (66% for **33b**) are obtained.

**Scheme 19:**
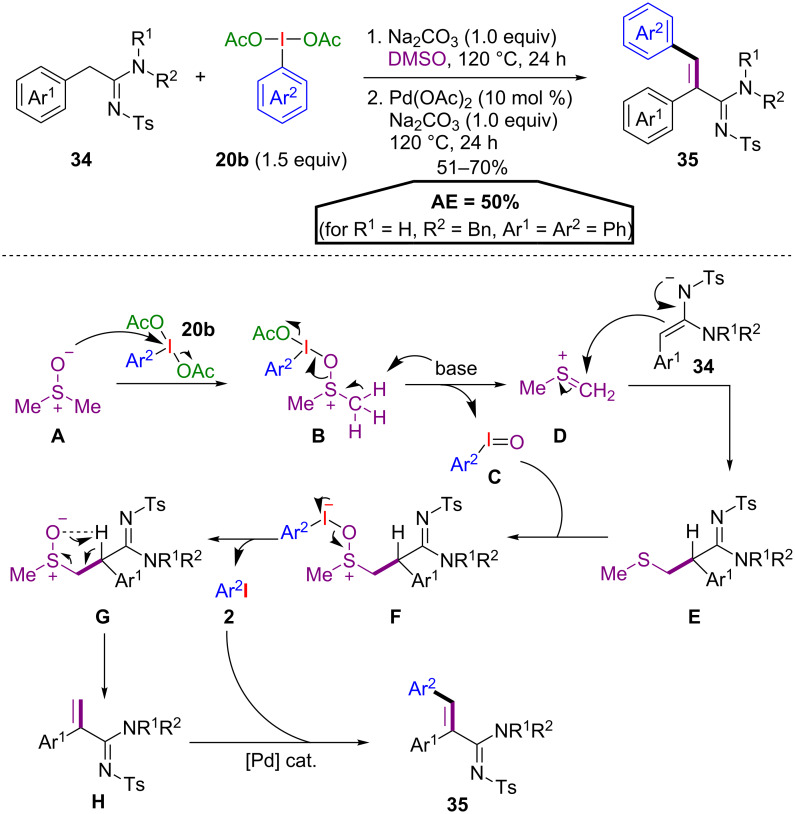
Tandem C(sp^3^)–H olefination/C(sp^2^)–H arylation.

In another tandem procedure, a metal-free C(sp^3^)–H olefination of amidines **34** with DMSO and PIDA derivatives **20b** was developed by Namitharan and co-workers [49]. A Pd(II)-catalysed Heck reaction of the formed exocyclic double bond with the emerging iodoarene yields substituted acrylamidines **35** (Scheme 19). The proposed reaction mechanism starts with the activation of DMSO (**A**) via the iodine(III) species **20b**. Iodosoarene **C** is released under basic conditions, forming the sulfonium intermediate **D**. This intermediate reacts with the amidine **34** to give the sulfide **E**, which is then oxidized to the sulfoxide **G** by iodosoarene **C** via the postulated activated sulfoxide **F**, releasing one equivalent of aryl iodide **2**. A Chugaev-type elimination yields the olefin **H**, which finally affords acrylamidine **35** via Heck coupling with the iodoarene **2** with 50% AE (for R^1^ = H, R^2^ = Bn, Ar^1^ = Ar^2^ = Ph).

### 3. Benziodoxolones

**Scheme 20:**
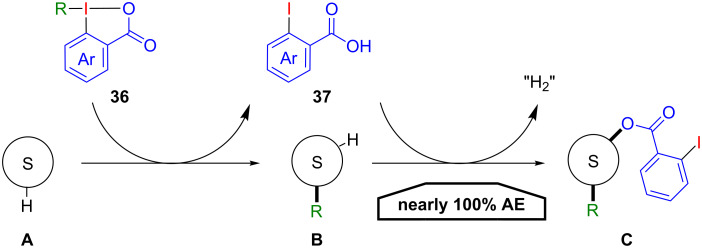
Atom efficient functionalisations with benziodoxolones **36**.

Iodine(III) compounds with a benziodoxolone or a benziodoxole structure are privileged reagents for electrophilic group transfer reactions, in particular in electrophilic alkynylations, azidations, cyanations and trifluoromethylations [3,50–54]. Here, 2-iodobenzoic acid is formed as a quantitative waste product, thus lowering the atom efficiency of these transformations. An atom-economical application of benziodoxolones **36** would involve the utilization of the nucleophilic properties of the released benzoic acid **37**, after the initial electrophilic group transfer to form **B** from substrate **A**. This would give target structures **C** that fully include the 2-iodobenzoic acid with a theoretical AE of nearly 100% (Scheme 20). Only molecular hydrogen is produced as formal waste in this process.

**Scheme 21:**
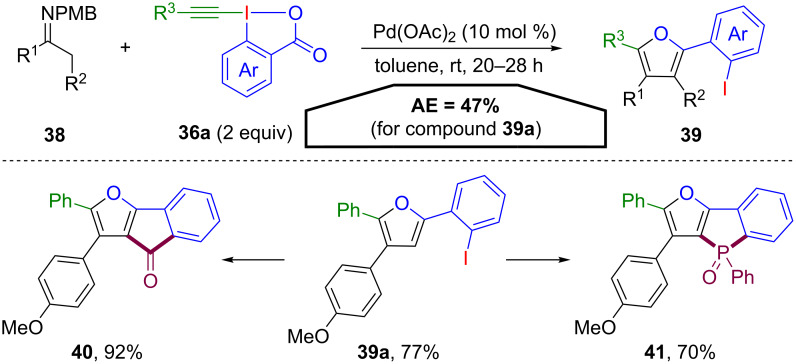
Atom-efficient synthesis of furans **39** from benziodoxolones **36a** and their further derivatisations.

The first report for an atom-economical application of benziodoxolones was a reaction of *N*-arylimines **38** with ethynylbenziodoxolones **36a** (EBX), under the influence of a palladium catalyst, developed by Yoshikai and co-workers [55]. Instead of the expected α-alkynylated product, highly substituted furans **39** were observed (Scheme 21). Both, the electrophilic alkyne and the nucleophilic 2-iodobenzoate group took part in this transformation in a complex putative reaction mechanism. However, since two equivalents of EBX (**36a**) are used, the AE for this transformation is rather low (47% for **39a**). The obtained furans **39** are highly useful synthetic intermediates, since the 2-iodoaryl group can be successfully employed in further derivatisations. In example, indenones **40** and phospholes **41** could smoothly be generated.

**Scheme 22:**
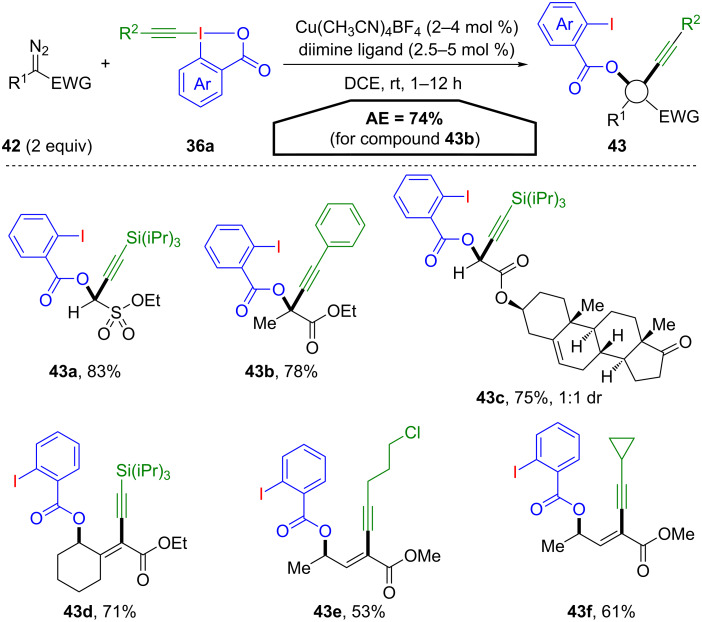
Oxyalkynylation of diazo compounds **42**.

In contrary to this unexpected reaction pathway, Waser and co-workers particularly aimed for an atom-economical application of EBX reagents **36a** utilizing a copper-catalysed reaction with diazo compounds **42** (Scheme 22) [56]. This strategy is not only atom economic regarding the applied hypervalent iodine reagent but also with regard to the chosen substrate. The metal carbene species generated from the diazo compounds displays nucleophilic as well as electrophilic reactivity at the same carbon atom and only gaseous dinitrogen is produced as stoichiometric waste. The reaction provides oxyalkynylated products **43** in high yields and addresses a broad scope of diazo derivatives **42** and EBX compounds **36a**. For diazo compounds bearing hydrogen, alkyl or aryl groups (R^1^), propargyl esters **43a**–**c** are obtained. In contrast, vinyldiazo compounds yielded the corresponding 1,3-enynes **43d**–**f** in good yields. The oxyalkynylation shows an excellent AE regarding the iodine(III) species **36a**. Every atom of the benziodoxolone **36a** is present in the final product **43** and only one equivalent of elemental nitrogen is lost over the course of the reaction. The overall AE is only diminished by the necessity to use two equivalents of the diazo compound (AE = 74% for **43b**).

**Scheme 23:**
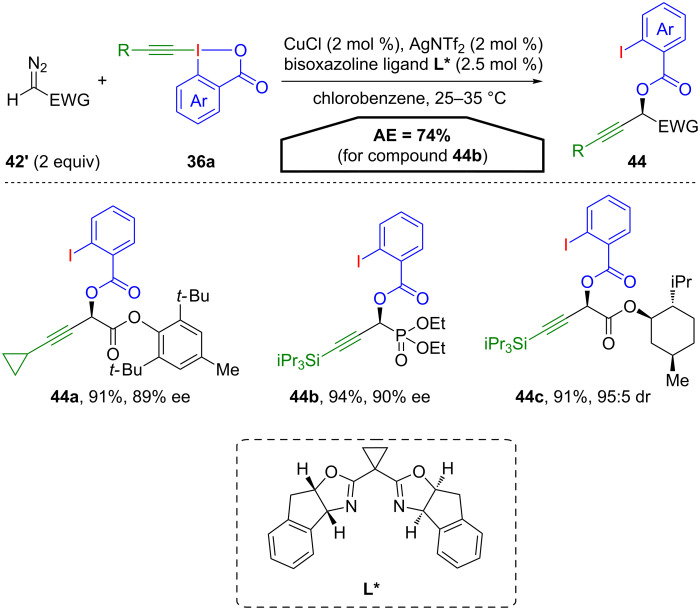
Enantioselective oxyalkynylation of diazo compounds **42’**.

Furthermore, an enantioselective version of this oxyalkynylation (for R^1^ = H) was developed [57]. Employing a chiral bisoxazoline ligand, diazo compounds **42’** with various electron-withdrawing groups were efficiently oxyalkynylated affording highly enantioenriched propargyl esters **44** (Scheme 23).

**Scheme 24:**
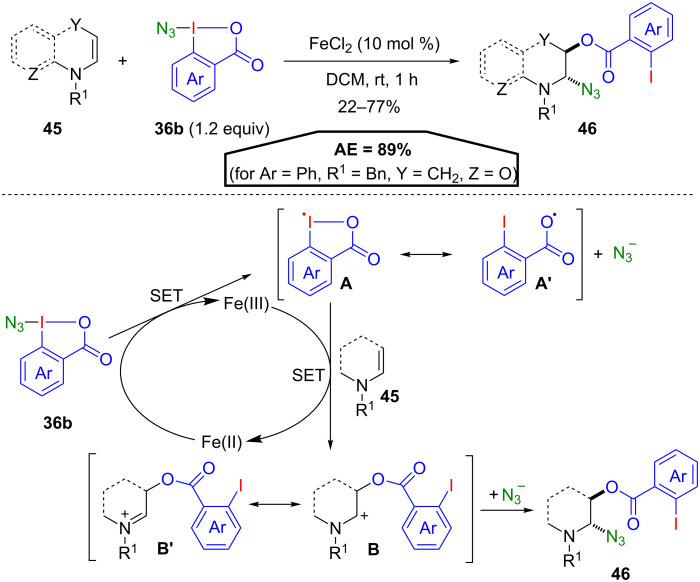
Iron-catalysed oxyazidation of enamides **45**.

Subsequent reductive cleavage of the ester gave direct access to enantiopure propargyl alcohols. Besides EBX reagents, azidobenziodoxolones **36b** (ABX), could be utilized for atom-economical reactions. Gillaizeau and co-workers developed an iron-catalysed oxyazidation of enamides **45** using ABX derivatives **36b** (Scheme 24) [58]. The reaction proceeds with complete regio- and stereoselectivity introducing the azide group in C2 and the ester moiety in C3 position, affording the *trans*-isomer **46** exclusively. The reaction mechanism presumably follows a radical pathway, which begins with a single electron transfer (SET) from Fe(II) to **36b** generating a Fe(III) species as well as benziodoxolonyl radical **A** or benzoyloxy radical **A’** and an azide anion. Next, a SET from the enamide **45** to Fe(III) affords again Fe(II) and a further reaction with the radical **A** or **A’** leads to the formation of carbocation **B** or iminium ion **B’**, respectively. Recombination of these positively charged intermediates with the azide anion finally affords the oxyazidated product **46**. Overall this oxyazidation exhibits the highest AE so far observed for iodane-mediated group transfer reactions with 89% (for Ar = Ph, R^1^ = Bn, Y = CH_2_, Z = O).

## Conclusion

In summary, we hope that we could demonstrate in this short focused overview, that aryl-λ^3^-iodanes are not only stable and highly reactive reagents for electrophilic group-transfer reagents, but that their utilization can be highly atom efficient if the emerging aryl iodides are constructively used in further transformations by cascade processes through a smart reaction design. The small number of discussable transformations, in which at least two of the three ligands attached to the central hypervalent iodine atom are transferred to a given substrate, exemplifies, that this field of research is still in its infancy. Nonetheless, this comparative article should give new impulses to researchers working in the field of iodane-mediated coupling reactions and hopefully will lead to new atom-efficient reaction methods utilizing these highly useful substrates, also in large-scale synthetic applications.
